# Patients’ Experiences of Telehealth in Palliative Home Care: Scoping Review

**DOI:** 10.2196/16218

**Published:** 2020-05-05

**Authors:** Simen A Steindal, Andréa Aparecida Goncalves Nes, Tove E Godskesen, Alfhild Dihle, Susanne Lind, Anette Winger, Anna Klarare

**Affiliations:** 1 Lovisenberg Diaconal University College Oslo Norway; 2 Palliative Research Centre Department of Health Care Sciences Ersta Sköndal Bräcke University College Stockholm Sweden; 3 Centre for Research Ethics & Bioethics Uppsala University Uppsala Sweden; 4 Oslo Metropolitan University Oslo Norway; 5 Clinical Psychology in Healthcare Department for Women’s and Children’s Health Uppsala University Uppsala Sweden

**Keywords:** health care technology, review, palliative care, telemedicine

## Abstract

**Background:**

Telehealth is increasingly being used in home care and could be one measure to support the needs of home-based patients receiving palliative care. However, no previous scoping review has mapped existing studies on the use of telehealth for patients in palliative home care.

**Objective:**

The aim of this study was to map and assess published studies on the use of telehealth for patients in palliative home care.

**Methods:**

A scoping review was conducted using the methodological framework of Arksey and O’Malley. Reporting was guided by Preferred Reporting Items for Systematic Reviews and Meta-Analyses extension for Scoping Reviews. A systematic and comprehensive search of Medical Literature Analysis and Retrieval System Online, EMBASE, PsycINFO, and Cumulative Index to Nursing and Allied Health was performed for studies published between January 2000 and October 2018. Two authors independently assessed eligibility and extracted data.

**Results:**

The review included 22 papers from 19 studies. Four thematic groupings were identified among the included papers: easy and effortless use of telehealth regardless of the current health condition, visual features that enhance communication and care via telehealth, symptom management and self-management promotion by telehealth, and perceptions of improved palliative care at home.

**Conclusions:**

The use of telehealth in palliative home care seems to be feasible, improving access to health care professionals at home and enhancing feelings of security and safety. The visual features of telehealth seem to allow a genuine relationship with health care professionals. However, there are contradicting results on whether the use of telehealth improves burdensome symptoms and quality of life. Future research should investigate the experiences of using telehealth among patients with life-limiting illness other than cancer and patients aged 85 years or older. More research is needed to increase the body of knowledge regarding the effectiveness of telehealth on symptoms and quality of life.

## Introduction

The preferred place of care for most patients in need of palliative care is their own home, and many of them are able to spend time at home and receive the needed care [1,2]. Patients want, as usual, to feel meaning and to maintain governance of their lives; thus, they prefer independent access to health care professionals when needed. Moreover, patients in need of palliative care express preferences for continuity of care and for health care professionals to provide coordinated care [2,3], which may be challenging in home care. Patients who receive care at home report unmet palliative care needs, such as the lack of regular communication with nurses and physicians and between primary and secondary health care professionals [4]. Furthermore, patients may feel uncertain about the urgency of their problems, whom they should contact in times of need, what the response may be, and the legitimacy of their needs, in addition to perceptions of poor continuity in home follow-up and coordination of services [4-6]. Physicians, nurses, physiotherapists, and social workers are often involved in the care and follow-up of patients in need of palliative care at home [7].

Telehealth is increasingly being used in home care [8] and could be one measure to meet the reported challenges and support the needs of home-based patients receiving palliative care. Telehealth is defined as “the provision of healthcare remotely by means of a variety of telecommunication tools” [8]. Telehealth can be delivered in an interactive mode, which invites an exchange of information or messages between patients and health care professionals, or in a passive mode, which is a form of communication that does not require an immediate response by the recipient [9].

Telehealth may be useful for conditions that require close monitoring, clinical assessment, and early intervention to prevent adverse events, such as unwanted emergency hospitalization [10]. The potential benefits of telehealth could include increased quality of life by improving independence and self-management with increased choice, improved access to community palliative care services for those wishing to die at home, and reduction in unnecessary hospital admissions [11]. Moreover, telehealth could be used to reduce patient travel burdens and provide access to services after regular clinic hours [12]. The use of telehealth appears to be promising as a help to meet patients’ expectations and needs related to maintenance of their care at home. However, challenges also exist. Head et al [13] claimed that palliative care has been characterized as high touch rather than high tech, which could limit the interest of health care professionals in applying technological advancements when developing and honing interventions [14].

Several systematic reviews have examined home-based telehealth in palliative care settings. One systematic review examined the evidence for home-based telehealth in pediatric care by including studies identified in two databases, focusing on children, adults, and health care professionals [14]. Other systematic reviews have examined patient outcomes [13] and caregiver outcomes [14,15] on telehealth interventions. Head et al [13] found heterogeneity regarding patient population and technology use. The outcome measures showed that all the included studies, except one, reported improvement in quality of life or symptom management. Another systematic literature review assessed the effectiveness of electronic health interventions for patients in palliative care or stakeholders such as health care professionals or caregivers [16]. Some of the included studies indicated positive results regarding quality of care, communication, and cost development. However, no randomized controlled trials (RCTs) were found.

Other literature reviews have been limited to geographical areas and have explored the use of telehealth in palliative care in the United Kingdom [17,18] and the development and use of mobile devices in palliative care services in sub-Saharan Africa [19]. In addition, the review by Johnston [17] was limited to older people. Kidd et al [18] found that telehealth was used by patients, relatives, and health care professionals in several contexts related to palliative care: oncology settings, specialist palliative care, primary care, and nursing homes. Telehealth applications including videoconferencing; consultations; symptom assessments; and advice for patients, relatives, and health care professionals were deemed usable and acceptable for patients and health care professionals.

Telehealth is increasingly being used in patients’ homes [8], and new technologies are being developed and implemented rapidly. Furthermore, as previously mentioned, some of the previous literature reviews are older and limited by geographical areas and to older patients. Consequently, there is a need to conduct an updated and broader literature review to develop an overview of the body of knowledge within this field and to identify gaps in knowledge for evidence-based practice. To our knowledge, no scoping review has mapped existing studies on the use of telehealth for patients in palliative home care. Consequently, this scoping review aimed to map and assess published studies on the use of telehealth for patients in palliative home care. The specific research question was as follows: What is known from the existing research literature about patients’ experiences of the use of telehealth in palliative home care?

## Methods

### Design

This scoping review used the framework of Arksey and O’Malley [20], which comprises five stages: identifying the research question; identifying relevant studies; study selection; charting the data; and collating, summarizing, and reporting the results. The reporting of the scoping review was guided by the Preferred Reporting Items for Systematic Reviews and Meta-Analyses extension for Scoping Reviews (PRISMA-ScR) checklist [21]. The protocol for this scoping review has not been registered or published.

### Identifying Relevant Studies

A systematic broad search was performed in October 2018 using the databases Medical Literature Analysis and Retrieval System Online (MEDLINE), PsycINFO, EMBASE, and Cumulative Index to Nursing and Allied Health (CINAHL) for studies published between January 1, 2000, and October 16, 2018. The search strategy was built in MEDLINE by 4 of the authors (SS, AAGN, AW, and AK) and a librarian using Medical Subjects Headings and text words. The search was adopted for each subsequent database. The search strategy is described in Multimedia Appendix 1. In addition, a hand search was performed to screen the reference lists of the included papers.

### Study Selection

On the basis of the inclusion and exclusion criteria (see Table 1), pairs of authors independently screened titles, abstracts, and full-text papers for inclusion in the study. When there was disagreement, an independent assessment of whether or not a publication met the inclusion criteria was conducted by a third author.

**Table 1:** Inclusion and exclusion criteria.

Criterion	Inclusion	Exclusion
Types of studies	Qualitative, quantitative, and mixed method studies on the phenomenon published in peer-reviewed journals	Letters, comments, conference abstracts, editorials, doctoral thesis, or any type of review
Period	January 1, 2000, until October 16, 2018	Before January 1, 2000, and after October 16, 2018
Language	English, Portuguese, Spanish, or Scandinavian	All other languages
Type of participants	Patients in a palliative care trajectory regardless of diagnosis, aged 18 years or older, and living at home	Patients who are not in a palliative care trajectory; patients aged 17 years or younger; and patients who use telehealth in a hospital, nursing home, or hospice setting
Phenomenon of interest	Patients’ experiences of using telehealth at home with follow-up from health care professionals	Patients’ experiences of using telehealth at home without follow-up from health care professionals or experiences of using telehealth in a hospital, nursing home, or hospice setting
Type of outcomes	Patients reported subjective and objective outcomes	Proxy-reported (next of kin or health care professional) outcomes

### Charting the Data

Pairs of authors extracted data from the included publications, using a standardized data-charting form and maintaining the wording and terminology from the papers. The form included the following information: authors, year of publication, country of origin, aim, population and sample size, telehealth application, delivered mode, design and method, and results (see Multimedia Appendix 2). Any disagreements were resolved by a third author.

### Collating, Summarizing, and Reporting the Results

An inductive approach was used to thematically organize and summarize the results from the included papers to answer the research question [20]. The extracted results from each paper were read several times to identify frequent patterns, similarities, and differences in patients’ experiences of using telehealth, regardless of the type of technology. The identified emerging patterns were organized in four thematic groupings. The first, second, and last author discussed the results and agreed upon the final groupings of the results. A frequency table illustrating which articles were included in which grouping was made (see Table 2).

**Table 2:** Articles included in thematic groupings.

Theme	Study	Number of articles
Easy and effortless use regardless of the current health condition	Aoki et al [22], Hochstenbach et al [23], McCall et al [24], Pinto et al [25], Whitten et al [26], Besse et al [27], Lind and Karlsson [28], Passik et al [29], Tieman et al [30], Reinke et al [31], Lind [32], Lind et al [33], Stern et al [34], Hennemann-Krause et al [35], van Gurp et al [36]	15
Visual features enhance communication and care via telehealth	Hebert et al [37], Miyazaki et al [38], Whitten et al [39], Whitten et al [26], Passik et al [29], Wilkinson et al [40], Reinke et al [31], Stern et al [34], Hennemann-Krause et al [35], van Gurp [36]	10
Symptom management and self-management promotion by telehealth	Bonsignore et al [41], Hebert et al [37], Hochstenbach et al [23], McCall et al [24], Miyazaki et al [38], Pinto et al [25], Whitten et al 30], Besse et al [27], Hoek et al [42], Lind and Karlsson [28], Wilkinson et al [40], Lind [32], Lind et al [33], Hennemann-Krause et al [35]	14
Perceptions of improved palliative care at home	Aoki et al [22], Bonsignore et al [41], Hochstenbach et al [23], McCall et al [24], Pinto et al [25], Whitten et al [39], Whitten et al [26], Lind and Karlsson [28], Wilkinson et al [40], Lind [32], Lind et al [33], Stern et al [34], van Gurp et al [43], van Gurp et al [36]	14

## Results

**Figure 1:**
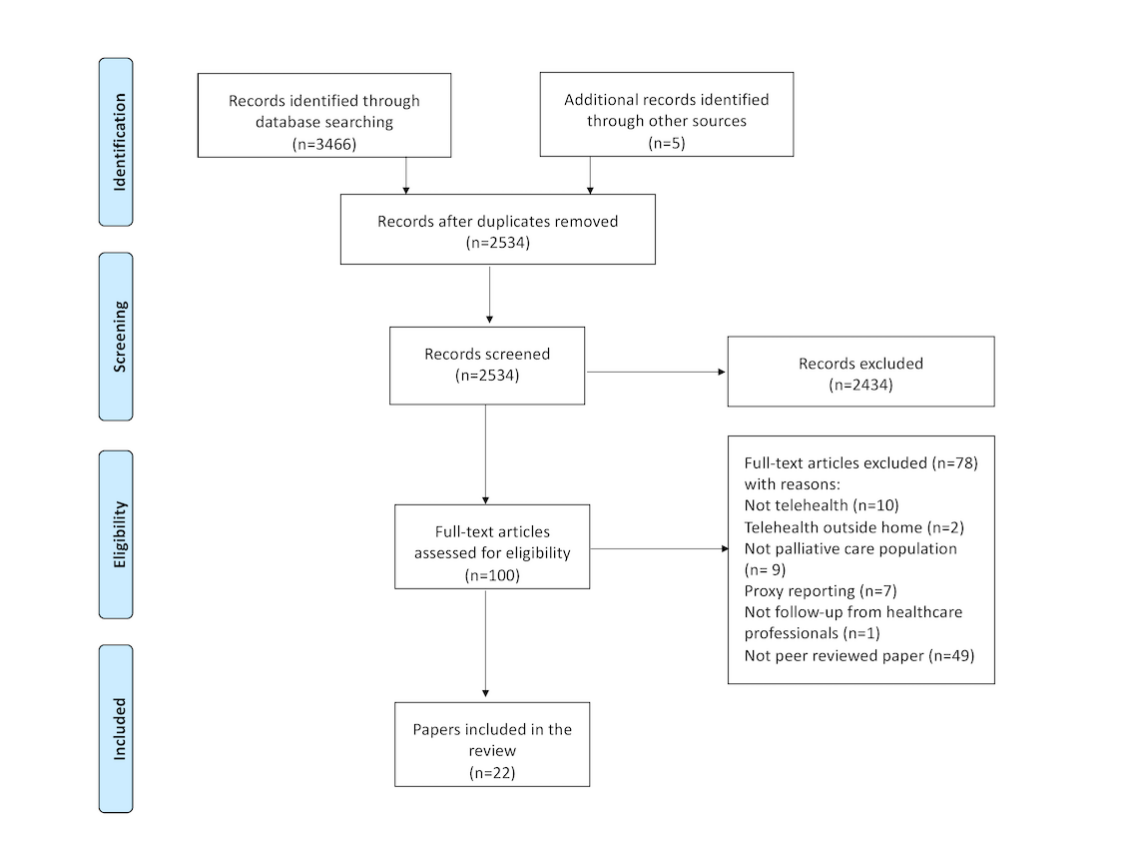
Summary of the selection of studies.

The database and hand searches yielded 3471 publications. After 937 duplicates were removed, titles and abstracts for 2532 publications were screened. On the basis of the inclusion and exclusion criteria, full text of 100 publications were read; 78 publications were excluded and 22 publications from 19 studies were included in the review (see Figure 1).

### Description of Included Studies

A total of 22 papers from 19 studies were included. The included studies were conducted in Australia (n=1), Brazil (n=1), Canada (n=2), Japan (n=1), the Netherlands (n=4), Portugal (n=1), Sweden (n=1), United Kingdom (n=3), and United States (n=5). The sample size of the included studies ranged from 2 to 187 participants, and in 6 papers, the samples consisted of 11 participants or fewer. Papers included patients with cancer (n=13); chronic obstructive pulmonary disease (COPD; n=1); cystic fibrosis (n=1); and a mix of different life-limiting illnesses such as cancer, COPD, multiple sclerosis, and amyotrophic lateral sclerosis (n=4). Three papers did not report diagnoses. Six papers included patients aged 85 years and above (oldest-old patients). Nine papers used mixed method or multimethod design, combining qualitative and quantitative methods [22-26]; 7 papers used quantitative design [27-31]; 3 papers used case study design [32-34]; 1 paper used case series design [35]; and 2 papers used qualitative design [36,43]. Three papers included an RCT [37,40,42]. The characteristics of the included studies are shown in Multimedia Appendix 2.

In 15 papers, telehealth was delivered using an interactive mode, whereas the passive mode was used in 7 papers (see Multimedia Appendix 2).

Video-based technology (n=14) was the most frequently used telehealth application in palliative home care. In 10 papers, teleconsultation with patients, relatives, and health care professionals was used to discuss patients’ needs, concerns, symptoms, and other problems and to give patients advice [22,26,30,35,36,39-42]. When possible, patients’ general practitioners participated from patients’ homes [36,42,43]. In four papers, videotelephone was used for individual contact between patients and health care professionals for support, symptom management [34,37,38], and dignity psychotherapy [29]. Webinar technology without video was used to enhance patients’ knowledge and skills about end-of-life issues and conversations [31].

Mobile devices such as mobile phones [27]; PDAs [24]; apps on smartphones, tablets, or PCs [23,25,41]; and digital pens and diaries [28,32] were used for pain education and the monitoring and management of pain and other symptoms.

Two studies described a theoretical framework for telehealth intervention: self-monitoring [23] and dignity psychotherapy [29].

To answer the research question regarding what is known about patients’ experiences of the use of telehealth in palliative home care, the results of this scoping review are presented in four thematic groupings: easy and effortless use of telehealth regardless of the current health condition, visual features that enhance communication and care via telehealth, symptom management and self-management promotion by telehealth, and perceptions of improved palliative care at home (see Table 2).

### Easy and Effortless Use of Telehealth Regardless of the Current Health Condition

Fifteen papers reported patients’ experiences of using the telehealth apps. In 10 papers, patients were able to use telehealth despite declining or poor health conditions. The apps were perceived as simple, clear, easy, effortless, and not too time consuming to use. Patients felt comfortable using the technology [23,25,27-34]. Although it was easy to use a digital pen and diary, because of the amount of information the patients received about the equipment, the diary, assessment, and the reporting of symptoms, the app was perceived as confusing [32,33]. App simplicity was seen as a prerequisite, especially for older people, in using the technology [22].

In four papers, patients were unable to use the apps because of their poor health condition, physical limitations, or unfamiliarity with the telehealth equipment, and they required assistance from their family [24,25,34,35]. Patients experienced challenges related to the design of the equipment, such as small font size on smartphones, the small size of videophones, or the lack of equipment portability [25,26,34,36]. The required use of a desktop device prevented teleconsultations for bedbound patients and reminded some of approaching death, which was not the case when the desktop device was replaced with a tablet device [36].

### Visual Features that Enhance Communication and Care via Telehealth

Patients experienced that telehealth including video was useful for communication and interaction with health care professionals [26,29,34-36,38-40]. Across these studies, patients noted that the visual features allowed them to see the health care professionals they were interacting with, which provided assurance and comfort as well as enhanced care and nursing assessments. The use of video enabled nonverbal communication, such as body language, facial expressions of happiness or suffering, and other emotions, in addition to the situational context [35,36]. According to van Gurp et al [36], this allowed patients and health care professionals to be immersed in a digital connectedness. Communication via video helped health care professionals to discern how patients felt, even when they tried to maintain a facade and pretend that everything was fine [36,39]. Moreover, a physically distant professional listener provided the freedom and privacy that patients needed to talk about difficult issues; thereafter, they continued as usual [36].

In 1 study in which patients were coached on end-of-life communication using webinars, patients reported that this format lacked interpersonal dynamics such as social presence as well as aural and visual communication cues that were considered important because of the sensitive nature of the topic. Nevertheless, patients stated that having the live video of the discussion facilitator and the ability to see other participants could have made it easier to follow the discussions [31]. In another study, although patients indicated a higher level of readiness to use video technology than home care nurses, patients preferred fewer visits overall and preferred to see the home care nurses in person [37].

### Symptom Management and Self-Management Promoted by Telehealth

There were equivocal results of whether the use of telehealth improved burdensome symptoms and quality of life. A study testing the feasibility of SMS and interactive voice response found a significant reduction in mean pain score using the European Organization for Research and Treatment of Cancer Quality Questionnaire, but the study found no change while using the Numeric Rating Scale for pain. Furthermore, there was no change in overall quality of life [27]. Another study, using TapCloud for remote monitoring of symptoms, found improved symptom management for moderate to severe dyspnea, moderate to severe depression, and poor well-being [41]. Two RCTs using teleconsultation found no significant differences between the telehealth group and the control group regarding symptom management and quality of life [37,40]. In contrast, an RCT investigating whether weekly teleconsultations from a hospital-based specialist palliative care team improved patients’ symptom burden found significantly higher symptom burden in the intervention group than the control group after 12 weeks [42].

Patients perceived that use of telehealth improved quality of care, enhanced self-management of pain, and contributed to more sincere pain reporting [23,26,28,32,38]. Patients who tended to forget which medication they had taken regarded the medication overview on the app as supportive, and those who took their analgesics based on the clock found the visual and sound reminders useful [23]. Patients perceived that the symptoms included in the app were too general [24], and they wanted the possibility to elaborate on their answers that were primarily related to pain, such as multiple localizations, type of pain, and why or how pain changed [24,33]. Furthermore, patients did not agree on how often the app for symptom monitoring should be used [25]. In 1 study, patients assessed symptoms using the Edmonton Symptom Assessment System, both at the hospital and at home via teleconferencing with a multidisciplinary team [35]. This study suggested that teleconferencing allowed improved symptom control.

### Perceptions of Improved Palliative Care at Home

Patients felt that the use of telehealth increased and improved their access to health care professionals at home [22-24,26,28,33,34,36,39,43] and perceived that they had increased access to health care professionals in case of emergency, during the night, or on an as-needed basis [26,39]. Furthermore, patients noted that contact with nurses was the most valuable component of the app [23].

It was important for the patients to know that health care professionals were available for them, were looking after them, and were monitoring them by means of telehealth. This contributed to feelings of being cared for at home, connectedness, relief, tranquility, and enhanced security [24,25,33,36,40]. Symptom monitoring at home facilitated communication of symptoms to hospital-based health care professionals [24,28,33], and remote home follow-up was perceived as less intrusive than a phone call [33,40]. During teleconsultations with their palliative care team and general practitioner, patients experienced concentrated responsiveness and possibilities for direct agreement on the division of responsibilities for future actions. However, patients felt insecure and needed to act as mediators when there was disagreement among health care professionals [43].

## Discussion

### Principal Findings

This scoping review mapped and assessed published studies on patients’ experiences of using telehealth in palliative home care. The results showed that telehealth apps seemed to be feasible for use in palliative care, increased and improved access to health care professionals at home, and enhanced feelings of security and safety. The visual features of telehealth allowed a close connectedness with health care professionals, although there were contradicting results on whether the use of telehealth improved burdensome symptoms and quality of life.

Telehealth apps seem to be feasible for use in palliative care and do not seem to add further burden to most patients. The prerequisites for patients’ willingness to use telehealth seem to be app simplicity and telehealth services being perceived as valuable to the patients [44]. However, the results indicate that the design issues of the apps had negative consequences of usability and user friendliness for some patients. Design issues may increase the dependency on help from others or act as a reminder of declining health and approaching death. Moreover, experiences of usability and other concerns related to telehealth may differ among patients with different illnesses [45]. To design and deliver apps and telehealth services that are even more closely aligned with patient needs, participation of users in the design process of technical solutions is crucial [46].

The results indicate that the use of telehealth improved access to health care professionals, although patients remained at their own homes. The use of telehealth seems to support the patient’s choice of living at home for as long as possible, which is important for many patients [2]. Patients may feel more comfortable, in control, and in a safer place at home compared with being in hospitals. In addition, the home environment seems more conducive to engaging in meaningful activities and relationships [2,36,47]. Patient choice and autonomy are essential in palliative care [48]. Promoting patients’ choice about *where* and *how* health care is provided may contribute to patients retaining stewardship over their lives, which is often compromised by illness [49].

The use of telehealth apps may strengthen the relationship between patients and health care professionals [50]. Our results suggest that in using telehealth, patients experienced a genuine relationship with health care professionals who looked after them and provided care according to their needs. This promoted feelings of being cared for and feeling secure. Trust is an important element of the health care professional-patient relationship, and it has been described as “a belief that good will be taken care of, or an attitude bound to time and space in which one relies with confidence on someone or something, and as willingness to engage in oneself in a relationship with acceptance of that vulnerability may arise” [51]. Trust may be regarded as something health care professionals must earn and work hard to attain. Availability and access to health care professionals and feeling physically and emotionally safe are described as important in this scoping review, in addition to respectful communication. These are some of the prerequisites for trust in the health care professional-patient relationship [51]. Patient awareness and the trust in the fact that health care professionals are watching over them may reduce feelings of loneliness with their health condition [52,53]. However, palliative care nurses have expressed concerns that technical issues with telehealth apps could jeopardize the relationship of trust with patients [54].

The finding that the use of video-based technology enhances communication and care is supported by previous reviews [55,56]. In line with a meta-ethnography on the experience of telehealth in patients with COPD [57], visual features using video images contributed to experiences of closeness, despite the remote contact. Although patients seem to highly value the use of telehealth, many simultaneously underline the importance and value of the physical presence of health care professionals at their home [58,59]. This may be especially important for those with limited social networks or poor social relations [2,57,59]. In addition, it may be more challenging to create a trusting relationship remotely than in person [8], and a caring touch, which patients in need of palliative care may appreciate [2], is impossible with remote contact. Some found that it may not be appropriate to discuss serious diagnoses or end-of-life issues via video because of the lack of physical closeness [60,61], whereas others found that the lack of physical closeness helped them address difficult issues with health care professionals. Consequently, health care professionals need to distinguish the appropriateness of using telehealth for communication and, in turn, individually tailor patient care [56,57].

Contradicting results were found on whether the use of telehealth improved burdensome symptoms and quality of life. Two one-group pre-post studies reported some improvements, whereas two of the RCTs found no significant differences between the groups. This is in line with a previous systematic review [13]. RCTs are considered the gold standard for investigating the effectiveness of interventions, as the design minimizes the risk of bias [62]. However, RCTs in palliative care research are often limited by poor recruitment, small sample size, and attrition, and consequently, quasi-experimental and observational design may be justified when randomization is considered inappropriate [63,64]. In the last RCT [42] included in this scoping review, a significantly higher symptom burden was reported in the experimental group compared with the controls. The patients in the experimental group may have had a higher awareness of symptoms leading to worsening symptom experience, or their symptoms may have been more precisely registered compared with controls, because of weekly teleconsultations [42]. Another explanation could be the increase in patients’ honesty in reporting their symptoms when using telehealth [32]. Nevertheless, this may also suggest that improving the symptom burden in these patients is complex.

A theoretical framework for understanding the mechanism of an intervention is recommended when conducting palliative care research on complex interventions [63]. Therefore, it was surprising that none of the studies that investigated whether the use of telehealth improved symptoms or quality of life applied such a framework. The use of theory in the development phase of the intervention is imperative to be able to explain eventual achieved effects [65]. The use of theory seems to be associated with positive results and large effect sizes [66].

Patients’ experiences of using telehealth in palliative home care have mostly been studied in populations comprising patients with cancer. Although the origins and the development of palliative care are closely linked to oncology, early integration of palliative care is increasingly emphasized [67,68]. Patients with life-limiting illnesses other than cancer also experience various problems and care needs early in the illness trajectories [69,70]. However, these patients may have a more unpredictable illness progression than patients with cancer, which presents a challenge in identifying the optimal time for introducing palliative care [71,72] and telehealth. However, patients with a life-limiting illness other than cancer may have been included in other studies, without the interventions being classified as palliative home care interventions.

Notably, few studies included the oldest-old patients, although this population increases continuously and also lives longer with life-limiting illness because of improvement in treatments [61,73]. There may be challenges to including the oldest-old patients in telehealth research. The oldest-old patients may not perceive telehealth as an appealing form of interaction with health care professionals, or health care professionals may inadvertently act as gatekeepers believing that old age and rapidly deteriorating health conditions make participation in telehealth research unfeasible [73].

This scoping review indicates that patients’ experiences of telehealth in palliative home care has mostly been studied in populations comprising patients with cancer, and few papers included the oldest-old patients. Mixed method is most frequently used for study design, whereas a limited number of papers used an RCT design. Furthermore, none of the papers that investigated whether the use of telehealth improved symptoms or quality of life applied a theoretical framework for their intervention.

A strength of this review was that we used an acknowledged framework for conducting scoping reviews, in addition to the PRISMA-ScR for guiding the reporting of the review. We performed a broad comprehensive and systematic search to identify published studies. Furthermore, the study selection process and data extraction were conducted independently by pairs of authors.

Considering the limitations of this review, different terms and synonyms are used for telehealth and palliative care in the literature [11,74]. There may be terms that we have not been able to identify and include in our search strategy. Patients with life-limiting illnesses other than cancer may have been included in studies without the intervention being classified as a palliative care intervention. Finally, our search strategy had language restrictions as we only included studies in English, Nordic, Spanish, and Portuguese. Owing to these choices, the results may be affected by information bias. Furthermore, only 6 of the 22 included papers were recent papers. Potential sources for heterogeneity in our scoping review are different study populations, diverse use of technologies, and different study designs across the included papers. Consequently, the results related to the patients’ experiences of telehealth should be interpreted with caution.

### Conclusions

The use of telehealth in palliative home care does not seem to add further burden to most patients. Telehealth increased and improved access to health care professionals at home, and it enhanced the feelings of security and safety. Furthermore, the visual features of telehealth allowed a close connectedness with health care professionals, which seemed to be highly valued. There were contradicting results on whether the use of telehealth improved burdensome symptoms and quality of life. The results further suggest that telehealth apps may be a positive addition to palliative home care, and patients’ reports thereof are in favor. However, health care professionals need to individually tailor the telehealth app to enhance usability and user friendliness for patients. Technology including video was preferable to patients.

To make solid inferences and suggest recommendations for practice and policy, more systematic reviews and studies highlighting the negative aspects of telehealth should be conducted. Future studies also need to address the experiences of using telehealth among patients with life-limiting illnesses other than cancer and the oldest-old patients. It is important to investigate whether other populations have different experiences of usability or other concerns regarding telehealth, as compared with patients with cancer and younger patients. Furthermore, studies including RCTs, when appropriate, are required to increase the body of knowledge regarding the effectiveness of telehealth on symptoms and quality of life. The involvement of users in the development of apps and studies is imperative. Using theoretical frameworks to better understand the mechanisms of interventions is important for future knowledge translation and application.

## Appendix

Multimedia Appendix 1 Search strategy used in Medical Literature Analysis and Retrieval System Online.

Multimedia Appendix 2 Characteristics of the included studies.

## Abbreviations

CINAHL: Cumulative Index to Nursing and Allied Health
COPD: chronic obstructive pulmonary disease
MEDLINE: Medical Literature Analysis and Retrieval System Online
PRISMA-ScR: Preferred Reporting Items for Systematic Reviews and Meta-Analyses extension for Scoping Reviews
RCT: randomized controlled trial
