# Supplementary material for: Patients’ Experiences of Telehealth in Palliative Home Care: Scoping Review
Source: J Med Internet Res. 2020 May 5;22(5):e16218. doi: 10.2196/16218 (PMC7238080; doi:10.2196/16218)
Supplement: Multimedia Appendix 1 [file jmir_v22i5e16218_app1.docx]

**Multimedia Appendix 1.** Search strategy used in Medline

| Search number | Mesh term, text word and combinations |
| --- | --- |
| 1 | Palliative Care/ |
| 2 | Palliative Medicine/ |
| 3 | "Hospice and Palliative Care Nursing"/ |
| 4 | exp Terminal Care/ |
| 5 | Terminally ill/ |
| 6 | (palliative adj2 (care or medicine or surgery or therapy or treatment* or rehabilitation or nursing*)).tw. |
| 7 | palliat*.tw. |
| 8 | (terminal* adj1 (ill* or care or treatment* or period)).tw. |
| 9 | hospice.tw. |
| 10 | "end of life".tw. |
| 11 | dying.tw. |
| 12 | exp TELEMEDICINE/ |
| 13 | exp VIDEOCONFERENCING/ |
| 14 | exp TELEREHABILITATION/ |
| 15 | exp After-Hours Care/ |
| 16 | Mobile Applications/ |
| 17 | exp Remote Consultation/ |
| 18 | Webcasts as Topic/ |
| 19 | TELECOMMUNICATIONS/ |
| 20 | Wireless Technology/ |
| 21 | exp Cell Phone/ |
| 22 | COMPUTERS/ |
| 23 | exp COMPUTERS, HANDHELD/ |
| 24 | (telemedicine or tele medicine or telehealth or tele health or telecare or tele care or telecommunicat* or tele communicat* or teleconferenc* or tele conferenc* or teleconsultat* or tele consultat* or telenursing or tele nursing or telemonitoring or tele monitoring or teletherap* or tele therap* or telerehab* or tele rehab*).tw. |
| 25 | (ehealth or e health or emedicine or e medicine or erehabilitation* or e rehabilitation* or mhealth or m health or ((electronic or mobile or digital) adj health)).tw. |
| 26 | (smartphone* or smart phone* or mobilephone* or mobile phone* or cellphone* or cell* phone* or personal digital assistant or palm pilot*).tw. |
| 27 | (health care technolog* or health technolog*).tw. |
| 28 | "after hours care".tw. |
| 29 | ((remote or internet or electronic or robot) adj1 (care or consultation*)).tw. |
| 30 | (assistive adj2 (living or technolog*)).tw. |
| 31 | 1 or 2 or 3 or 4 or 5 or 6 or 7 or 8 or 9 or 10 or 11 |
| 32 | 12 or 13 or 14 or 15 or 16 or 17 or 18 or 19 or 20 or 21 or 22 or 23 or 24 or 25 or 26 or 27 or 28 or 29 or 30 |
| 33 | 31 and 32 |
| 34 | limit 33 to yr="2000 - 2018" |
