# Supplementary material for: Patients’ Experiences of Telehealth in Palliative Home Care: Scoping Review
Source: J Med Internet Res. 2020 May 5;22(5):e16218. doi: 10.2196/16218 (PMC7238080; doi:10.2196/16218)
Supplement: Multimedia Appendix 2 [file jmir_v22i5e16218_app2.docx]

**Multimedia Appendix 2.** Characteristics of the included studies.

| **Author, year,**  **Country** | **Aim** | **Sample** | **Telehealth application, delivered mode** | **Design and methods** | **Results** |
| --- | --- | --- | --- | --- | --- |
| Aoki et al. [22]  2006  Japan | To describe experiences of implementation tele-palliative care in rural community for patients who want to receive home-based palliative and to conduct qualitative and quantitative evaluations of tele-palliative care to investigate is clinical and economic impact | 2 patients (cancer); 1 female, age 72 and 78 years | *Teleconference* with physician, palliative care staff, patient, caregiver. Conversation (symptoms, questions, concerns), recording of vital signs, advices to patient and caregiver. Patients and caregivers could contact the palliative care unit 24h / 7d in case of emergency using the system. Interactive mode | Triangulation method  Semi-structured interviews  Quantitative analysis of a situated model for tele-palliative care | Patients believed tele-palliative care could increase access to clinical service. Usability was an important area to improve. Home care was the most cost-saving strategy if the care was continued for more than 4 months. The number of physician visits during tele-palliative care could be reduced to less than 6 physician visits per month |
| Besse et al. [27] 2016  The Nederlands | To perform a feasibility study on the use of short message service (SMS) and interactive voice response (IVR) to improve pain management in patients with cancer, including terminally ill patients | 13 patients (cancer); 3 females, age 27-75 years, 12 palliative treatment | During 4 weeks, 4 days per week, twice per day, patients received a daily IVR asking to provide their pain score on a NRS^1^ with their mobile phone. If NRS score > 4 a nurse contacted the patient the same day and if necessary adapted pain treatment. Passive mode | One group pre- and post test | Application was easy to use, did not interfere with privacy, was not burdensome, or too time consuming. No significant change in NRS pain score was found. Score on the EORTC QLQ-C30^2^ pain scale decreased significantly (p 0.047) |
| Bonsignore et al. [41] 2018  USA | To describe a telehealth palliative care program using the TapCloud (TC) remote patient monitoring application and videoconferencing; evaluate the feasibility, usability, and acceptability of a telehealth system in palliative care; and use a quality data assessment collection tool in addition to TC ratings of symptom burden and hospice transitions | 101 patients (23 pulmonary, 19 cancer, 14 neurological, 14 cardiovascular, 5 renal, 3 gastrointestinal, 23 others); 61 females, mean age 72 years | TC application for remote patient monitoring (wellbeing, pain, symptom and medication management, messaging, photo upload). Palliative care team sent push messages to patients via application to remedy situation. *Videoconferencing* was used to meet patients need, if necessary, a home visit was arranged.  Interactive mode | Mixed methods approach  Quantitative assessments  Semi-structured interviews | Remote patient monitoring using TC resulted in improved symptom management. 35 % were transitioned to hospice. TC gave patients a deep sense of comfort, knowing that a qualified care team was only a tap away. Patients perceived that TC was not able to replace the depth of in-person care |
| Hebert et al. [37] 2006  Scotland | To compare conventional palliative homecare visits and a combination of conventional and home telehealth ‘video-visits’ with respect to symptom management and quality of life | Intervention Group (IG): 25 patients (diagnosis NR); 16 females, mean age 70 years (range 43-98)  Control Group (CG): 19 patients (diagnosis NR^3^); 12 females, mean age 63 years (range 36-94) | IG: Combination conventional and home telehealth video visit, NR who followed-up patients  CG: Conventional palliative care home visit  Interactive mode | Multi-method study  Randomised Control -trial (RCT)  Semi-structured interviews | No significant differences were found between the groups in measure of symptom management and quality of life. Patients indicated higher level of readiness to use the video technology than nurses. Patients preferred fewer and in person visits overall. Factors influencing the patients’ preferences included the waiting time for unplanned visits |
| Hennemann-Krause et al. [35] 2015  Brazil | To examine telemedicine as a form of home and additional support for traditional outpatient care as a way to remotely monitor and manage the symptoms of patients with advanced cancer | 12 patients (cancer); 5 females, mean age 68 years Standard Deviation (SD) =9.43 | Monthly in-person consultations at hospital and weekly web conferences with multidisciplinary team (MT) (physician, nurse, social worker, psychologist and music therapist) at home. Patients received home visits when necessary. Communication between MT and patients was also performed by email and telephone calls. ESAS^4^ was applied during all remote and in-person visits. Interactive mode | Prospective, longitudinal, qualitative, descriptive, and case series study | Patients were not able to use the telehealth equipment by themselves, due to unfamiliarity with the equipment. Patients were not able to use ESAS when clinical condition worsened. The 8 patients who completed the ESAS in all interviews had lower mean distress symptom scores according to web conferences than in-person consultations |
| Hochstenbach, et al. [23] 2016  The Netherlands | To explore feasibility of the mobile application for patients, the web application for nurses, and the integration of both applications in routine clinical practice | 11 patients (cancer) with moderate to severe pain under palliative anti-tumour treatment (n=11); 6 females, mean age 53 (range 20-76, SD=15) years | Web application for nurses. Mobile application for patients containing pain diary, personalized medication schedule and pain education. Communication between nurses and patients via text messages using the mobile application and the web application for nurses. Passive mode | One-group post-test  Questionnaires  Semi-structured interviews | Patients quickly learned how to manage the application, easily used the different components and liked to work with the application Average completion rates were 76% for pain monitoring, 50% for medication monitoring and 100% for education sessions |
| Hoek et al. [42] 2017  The Netherlands | To determine whether weekly teleconsultations from a hospital-based specialist palliative care consultation team (SPCT) improved patient-experienced symptom burden compared to “care as usual” Secondary objectives were to determine the effects of these teleconsultations on unmet palliative care needs, continuity of care, hospital admissions, satisfaction with teleconsultations | IG: 38 patients (cancer); 11 females, mean age 62 years (SD=9)  CG: 36 patients (cancer); 14, mean age 62 years (SD=11) | IG: Weekly teleconsultations for a 12-week period with a SPCT member (nurse or physician) using desktop/tablet was added to usual care. General practitioner (GP) invited to join patient during the teleconsultations. Teleconsultations covered all domains of palliative care.  CG: Usual care: palliative homecare provided by their GP, supported by the SPCT according to the standard referral procedure by phone or patient visit to outpatient clinic. Interactive mode | RCT | Significant higher symptom burden in IG compared with the CG at week 12 (adjusted difference 6.90; 95% CI 0.17-13.63, p 0.04). The adjusted anxiety scores were higher in the intervention group than controls (estimated effect 1.40; 95 % CI 0.12-2.55, p 0.03). No difference between groups regarding depression, unmet needs, experienced continuity of care and reported hospital admissions |
| Lind [32] 2008  Sweden | To explore and describe palliative home care patients’ experiences of assessing their pain by using paper pain diaries together with digital pen and mobile internet technology | 12 patients (cancer); sex and age NR | Patients completed pain assessments 3 times per day using a digital pen and pain diary form in digital paper*.* Nurses or physicians viewed the  assessments at the clinic and used the data in their medical decisions. Passive mode | Descriptive and explorative case study | Patients perceived that they took greater part in own care, in addition to improved and increased contact with their healthcare professionals. The digital pen was easy to use, and the pain diary was easy to interpret |
| Lind & Karlsson [28]  2004  Sweden | To develop a system that can improve today’s information and communication techniques within home healthcare, and that will contribute to improving the quality of home healthcare | 12 patients (cancer); sex and age NR | Patients completed pain assessments 3 times per day using a digital pen and pain diary form in digital paper. Nurses or physicians viewed the assessments at the clinic and used the data in their medical decisions. Passive mode | Questionnaire study | Patients experienced increased caregiver contact and that the technology positively affected the quality of care. The digital pen was easy to use, and pain diary was easy to understand |
| Lind et al. [33]  2008  Sweden | To explore and describe palliative home care patients’ experiences of assessing their pain by using a pain diary together with digital pen and mobile internet technology | 12 patients (cancer) with VAS^5^ pain ≥ 35 mm; 4 females, mean age 67 (range 58-79) years | Patients completed pain assessments 3 times per day using a digital pen and pain diary form in digital paper containing VAS (0-100 mm). Nurses or physicians viewed the assessments at the clinic, gave patients feedback on received assessments and used the data in their medical decisions. Passive mode | Case study  Interviews, questionnaire, patient medical records, pain management system | Pain assessment method was effortless despite of patients’ state of health, improved contact with caregivers, increased participation in own care and sense of security. The equipment was easy to use |
| McCall et al.  [24] 2008  UK | To test the acceptability and usability of the Advanced Symptom Management System in Palliative Care (ASyMSp) | 21 patients (cancer); females 7, mean age 64 (range 47-87) years | Using mobile phone-based technology, AsyMSp, to remote monitor and manage patients’ symptoms including symptom graphs and relevant self- care advice, completed on daily basis, or when patients felt unwell. Patients’ assessments were immediately available to healthcare professionals. Passive mode | Explorative, descriptive  Questionnaire  Semi-structured interviews | All patients were comfortable using technology, some needed help from family. Patients perceive the tool as helpful to manage and to communicate symptoms to staff but wanted to explain their responses, especially with pain. System helped them feel cared for and looked after |
| Miyazaki et al. [38] 2003  Canada | To examine the usability of an ISDN-based videophone (VP) for home care | 4 patients (diagnosis NR); 4 females, age NR | Staff (nurse, respiratory therapist) support via VP was available for average of six weeks, in the evenings but not weekends. Installation of technology and training session on use given in home was performed. Interactive mode | Multi-method  Semi-structured interviews  Questionnaire | VP provided patients with comfort and reassurance. Visual features enhanced care, comforting to see professional when distress and possibility to receive support in presence of distress. |
| Passik et al. [29] 2004  USA | To determine if telemedicine is a feasible way to make Dignity Psychotherapy available to patients who are dying at home | 8 patients (cancer) with life expectancy < 6 month; 2 females, mean age 56 (range 41-66) years | Dignity Psychotherapy sessions given by palliative care staff, through videophone bolster desire to live, lessen desire to die and improve quality of life. Sessions were recorded, transcribed and returned to patient as legacy. Interactive mode | Feasibility study  Questionnaires | Patients were largely comfortable with videophone technology and would not necessarily have preferred to engage in face-to-face sessions |
| Pinto et al. [25] 2017  Portugal | To introduce a web-based application for monitoring comfort in patients receiving palliative care | 7 patients (cancer 4, ALS^6^ 2, MS^7^ 1) with life limiting illness; 2 females, mean age 41 (range 27-54) years | The comfort app was compatible with smartphone, tablets or computer. Included contact information with a palliative care (PC) team, had a message function and assessed 11 self-reported physical and phycological items (0-10). The PC team was informed by a researcher if the item score ≥ 4. Passive mode | Multi-phase electronic development project; feasibility and acceptability assessment | The app was simple, clear, easy to use and considered useful. Some patients preferred to access app using computer due to physical limitations. Knowing that someone was looking out for them was experienced as important. All items were considered appropriate. Opinions differed of how often symptoms should be rated |
| Reinke et al. [31] 2011  USA | To test the feasibility and preliminary efficacy of an interactive web-based seminar to equip patients with COPD^8^ with the knowledge and skills to engage in conversations about their end- of- life issues | 7 patients (COPDstage III-IV); 7 females; age 68 (SD=4) years | Web conference technology: Webinar with pictures of facilitator (nurse) and research assistant (nurse); attendee list and chat function. Technology training and open group sessions to meet and greet before webinar. Link to webinar mailed out with article of end-of-life issues to discuss; webinar lasted 60 minutes. Interactive mode | One-group pre-post test | Patients felt that technology was easy to use, but the conversation dynamics were difficult and lacked interpersonal dynamics. 3 months after the start of the intervention, all patients had experienced some form of action and communicated end-of-life wished to their family or clinician |
| Stern et al. [34] 2012  Canada | To explore family caregivers and palliative cancer patients’ patterns of use, perceptions of, and experiences with home telehealth | 11 patients (cancer); 4 females, median age 61 (range) 34–91) years | Telephone or videophone communication. Devices for remote monitoring of blood pressure, oxygen levels, heart, lung and abdominal sounds. Specialist nurses were available 24 hours per day who communicated with patients and families using videophones, with optional remote monitoring. Interactive mode | Mixed-methods case study  Computerized nursing documentation  Chart abstraction  Interviews | Patients described that equipment was easy to use. All needed assistance with blood pressure cuff application. They were able to operate pulse oximeter and videophones independently. Visual access to care provided reassurance. |
| Tieman et al. [30] 2016  Australia | To investigate the feasibility of a telehealth-based model of service provision for community based palliative care patients, carers and clinicians | 43 patients (41) cancer) with AKPS^9^ > 40;17 females, average age 72 (range 49 to 91) years | Video-based conferences between  service staff (nurses) and patient or carer.  Virtual case conferences between patient and carer, service staff and patient’s general practitioner. Self-report assessment tools for the patient and carer, and remote activity monitoring. Interactive mode | Prospective cohort study | Patients were able to use the technology and did self-report using applications. Pain and fatigue were the most self-reported symptoms. On average patients self-reported 25% more frequently than expected |
| van Gurp et al. [43] 2016  The Netherlands | To describe whether and how  teleconsultation supports the integration of primary care, specialist palliative care team (SPCT), and patient perspectives and services and how patients and (in)formal caregivers experience collaboration in a teleconsultation approach | 18 patients (16 cancer, 2 COPD) with life expectancy < 3 moths; 8 females, mean age 61 (range 24–85) years | Teleconsultation, using desktop computer or tablet*,* between hospital based SPCT (physicians, nurses), patients, families and primary care physician (PCP) that was added to the existing community care model. Interactive mode | Qualitative  Observations  In-depth interviews | Patients could start favour teleconsultations with SPCT over PCP because of increased connectedness with SPCT. In tri-partite teleconsultations, patients experienced a concentrated responsiveness and opportunities for direct agreements on responsibility for future action |
| van Gurp et al. 2015 [36]  The Netherlands | To examine how telemedicine facilitates or limits the patient-professional relationship, which is considered the key to palliative care | 18 patients (16 cancer, 2 COPD) with life expectancy < 3 moths; 8 females, mean age 61 (range 24–85) years | Weekly synchronous audio-visual videoconferencing, using desktop computer or tablet were performed between SPCT (physicians, nurses), PCP and patients. Patients reported symptoms and multi-dimensional problems. Healthcare professionals provided practical advice. Interactive mode | Qualitative Observations  Semi-structured and open interviews | Teleconsultations enabled specialized palliative care at home, obviating distressing hospital admissions. Visual features digital connectedness, exposed discrepancies in patients’ stories. Teleconsultations created feeling of safety, intimacy and relief. Desktops reminded some patients of approaching death |
| Whitten et al.  [26] 2004  USA | To examine telephospice from the patient’s perspective and to addressee use of services and patient acceptance | 187 patients (114 cancer, 73 other diagnosis); 95 females, mean age 71 (range 26-98) years | Use of videophones that transmitted real-time video and audio images through analog phone system. Patients and their caregivers received traditional hospice services supplemented with nursing, social work, and spiritual care tele visits from nurses, social workers, and spiritual care providers. Interactive mode | Multi-method  Survey  In-depth interviews | Patients initiated a call less than 1 % of the time. Care via telephospice was different due to lack of physical proximity which could be beneficial. Best thing with telephospice was immediate access to providers which provides comfort. Some wanted to change some aspect of equipment |
| Whitten et al.  [39] 2001  USA | To provide end‐of‐life services to hospice patients who lived at home and their caregivers by videophones and examine a wide array of issues from provider and patient perspectives, specifically: costs, access, delivery and satisfaction | 56 patients (diagnosis NR); sex and age NR | Telephospice (TH): Use of videophones for multidisciplinary team (i.e., nurses, physicians, social workers, and spiritual care providers) for consultation and support to palliative patients who decide to live at home until the end of their life if possible. Interactive mode | Multi-method  Survey  Open-ended interviews | 33% of the patients perceived that TH increased their access to their Healthcare professional. Patients felt that TH facilitated instant face-face communication, improved traditional hospice care. Many desired to utilize the TH equipment for more daily hospice procedures and the equipment was perceived as safe and effective |
| Wilkinson et al. [40]  2008  UK | To investigate the feasibility of a video link to support patients on the transplantation waiting list and their families | 7 patients (cystic fibrosis; 3 females, median age 28 (22-38) years.  CG: 4 patients (cystic fibrosis); 1 female, median age 26 (21-29) years | IG: Videoconferencing unit via ISDN connected to television; micro-spirometer, pulse-oximeter, single use clinical thermometers. Contact on a weekly basis with nurses, physiotherapist or other healthcare professional if needed. Non-invasive ventilation, physiotherapy, sputum, mobility, nutritional status and other problems were discussed. CG: Usual care NR.  Interactive mode | RCT | No significant differences in quality of life, anxiety levels, depression levels, admissions to hospital, clinic attendances, general practitioner calls and intravenous antibiotic use between the two groups after six months were found. A significant improvement in perception of body image for IG. Patients liked and valued the service |

^1^Numeric Rating Scale; ^2^EORTC QLQ-C30: European Organization for Research and Treatment of Cancer Quality of Life Questionnaire; ^3^NR: not reported; ^4^ESAS: Edmonton Symptom Assessment System; ^5^VAS: Visual Analog Scale; ^6^ALS: amyotrophic lateral sclerosis; ^7^MS: multiple sclerosis; ^8^COPD: Chronic Obstructive Pulmonary Disease; ^9^AKPS: The Australia-modified Karnofsky Performance Scale
